# ‘Moving towards understanding’, acceptability of investigations following stillbirth in sub‐Saharan Africa: A grounded theory study

**DOI:** 10.1111/1471-0528.17319

**Published:** 2022-10-21

**Authors:** Carol Bedwell, Valentina Actis Danna, Kutemba Lyangenda, Khuzuet Tuwele, Flora Kuzenza, Debora Kimaro, Happiness Shayo, Chisomo Petross, Isabella Chisuse, Alexander Heazell, Suresh Victor, Bellington Vwalika, Tina Lavender

**Affiliations:** ^1^ Centre for Childbirth, Women’s and Newborn Health International Public Health, Liverpool School of Tropical Medicine Liverpool UK; ^2^ Department of Public Health and Research Ministry of Health Lusaka Zambia; ^3^ Archbishop Antony Mayala School of Nursing Catholic University of Health and Allied Sciences Bugando Mwanza Tanzania; ^4^ Kamazu College of Nursing University of Malawi Blantyre Malawi; ^5^ Faculty of Biology, Medicine and Health University of Manchester Manchester UK; ^6^ Perinatal Imaging and Health King’s College London London UK; ^7^ School of Medicine University of Zambia Lusaka Zambia

**Keywords:** autopsy, investigations, postmortem, stillbirth, sub‐Saharan Africa

## Abstract

**Objective:**

To explore the views of women, partners, families, health workers and community leaders of potential investigations to determine the cause(s) of stillbirth, in Malawi, Tanzania and Zambia.

**Design:**

Grounded theory.

**Setting:**

Tertiary facilities and community settings in Blantyre, Malawi, Mwanza, Tanzania and Mansa, Zambia.

**Sample:**

Purposive and theoretical sampling was used to recruit 124 participants: 33 women, 18 partners, 19 family members, 29 health workers and 25 community leaders, across three countries.

**Methods:**

Semi‐structured interviews were conducted using a topic guide for focus. Analysis was completed using constant comparative analysis. Sampling ceased at data saturation.

**Results:**

Women wanted to know the cause of stillbirth, but this was tempered by their fear of the implications of this knowledge; in particular, the potential for them to be blamed for the death of their baby. There were also concerns about the potential consequences of denying tradition and culture. Non‐invasive investigations were most likely to be accepted on the basis of causing less ‘harm’ to the baby. Parents’ decision‐making was influenced by type of investigation, family and cultural influences and financial cost.

**Conclusions:**

Parents want to understand the cause of death, but face emotional, cultural and economic barriers to this. Offering investigations will require these barriers to be addressed, services to be available and a no‐blame culture developed to improve outcomes. Community awareness, education and support for parents in making decisions are vital prior to implementing investigations in these settings.

## Abstract 2

**Linked article**: This article is commented on by Elizabeth M. McClure and Robert L. Goldenberg, pp. 68‐69 in this issue. To view this mini commentary visit https://doi.org/10.1111/1471‐0528.17310.

## 1 INTRODUCTION

Women who have experienced stillbirth have expressed the need to understand the reasons for their baby’s death.^1^, ^2^ Of the 820 000 stillbirths per year in sub‐Saharan Africa,^3^ at least a quarter are unexplained.^1^, ^4^ The limited availability of postmortem investigations leaves women and families without the answers they require to process their grief. The absence of investigations and a cause for stillbirth can impact on women’s psychological wellbeing and on the ability of health professionals to provide appropriate targeted care in future pregnancies.^5^ In addition, failure to understand the cause of stillbirth leaves women open to stigma and blame.^6^ Receiving and understanding autopsy results has been associated with better emotional outcomes for women, as they are able to gain closure and are absolved of blame.^7^, ^8^

Reducing stillbirth is a health priority driven by Sustainable Development Goal (SDG) 3, and global policy, including Every Newborn Action Plan^9^ and the Global Strategy for Women’s Children’s and Adolescents’ Health;^10^ which aim to reduce the stillbirth rate to fewer than 12 per 1000 live births and end preventable stillbirths by 2030. However, without understanding the reasons for stillbirth, these goals will be difficult to reach. This is a particular issue in sub‐Saharan Africa, which continues to have rising rates of stillbirth and the highest stillbirth burden globally.^3^

There is limited access to investigations in low‐ and middle‐income countries (LMICs) such as Malawi, Tanzania and Zambia, despite the high burden of stillbirth in the Region.^3^ Where investigations are available, the financial cost is met by the parents. In many cases, clinical records and verbal autopsy are the only means of determining cause, but these rely on clinician judgement and are subject to misclassification.^1^, ^4^, ^11^ Full autopsy investigations require highly trained staff and additional costs, which may make them unviable in some LMICs, where resources are already stretched.^11^ Even if available, cultural expectations, practices and beliefs may prevent women from accessing these services.^5^ Moreover, the costs of autopsy may prove prohibitive to parents. Non‐invasive or minimally invasive autopsy may be more culturally acceptable to parents^5^ and place less of a burden on health systems. Such investigations include biopsy, placental examination and histology, imaging including magnetic resonance imaging (MRI) or computed tomography (CT) and verbal autopsy.^11^, ^12^, ^13^, ^14^ Some methods, such as MRI or CT imaging, have been reported to be as effective as a conventional postmortem.^13^ The aim of this study was to explore the views of women, partners, families and stakeholders in relation to the type of investigations that would be culturally acceptable, if any, and how the consent process should be approached.

## 2 METHODS

A Straussian grounded theory approach was taken;^15^ this allowed for the understanding of participants’ views and experiences though social interaction. Participants were recruited primarily from similar public tertiary facilities in Blantyre, Mwanza and Mansa in Malawi, Tanzania and Zambia, respectively. Tertiary facilities were selected, as the majority of women experiencing complications resulting in stillbirth will have been transferred to this level of care during pregnancy or labour. This included women from semi‐urban and rural areas to allow for a variety of views. Furthermore, these sites were partners in a programme of work aimed at preventing and managing stillbirth, funded by NIHR (16/137/53). Community Engagement and Involvement (CEI) groups, consisting of women who had themselves experienced stillbirth, further identified local women, families and stakeholders in the community for potential recruitment to the study. Women were included if they were over 18 years, had the capacity to consent and had experienced a stillbirth at 28 weeks’ gestation or later, as per the World Health Organization (WHO) definition,^3^ as adopted by the included countries. Women were identified by health workers, who made the initial approach and collected consent to contact forms from women interested in taking part. Women who agreed to contact were then approached by the researcher. Partners and family members of consenting women were approached by the researcher if women gave permission for this. Community leaders were identified by CEI group members in the community. Those interested in participating were introduced to the researcher by a CEI member. Health workers opted into the study using contact details on advertisements in the clinical environment. Sampling commenced with purposive sampling of three participants of each of the following groups: women who had experienced a stillbirth, partners and family members of women experiencing stillbirth, health workers working in maternity wards and community leaders. Theoretical sampling continued until data saturation was confirmed, when no new themes emerged from the data.^16^ Participants were recruited by Lugina Africa Midwives Research Network (LAMRN)‐trained researchers, ^17^ with experience in taking informed consent and qualitative interviewing. Health workers were recruited via posters and information inviting them to participate in the study.

Interviews were conducted in a mutually convenient setting; home, facility or community building, and were audio‐recorded with consent. Women, partners and family members were interviewed in the local language, with health professionals and community leaders choosing their preferred language. Interviews were respondent‐led, with topic guides used to maintain focus. A description of potential investigations, including biopsy, skin swab, placental investigation and postmortem was provided by the interviewing research assistant using a standardised format (Table S1). Demographic data were collected as part of the interview to enable contextualisation of the data. A summary of the interview was confirmed with participants by the researcher, confirming validity.

Constant comparative analysis of the data guided by Strauss & Corbin^15^ was completed by in‐country researchers (by country) and synthesised/integrated by UK leads. Transcribed interviews underwent translation and back‐translation to ensure accuracy. Transcripts were read in their entirety for familiarity and open, axial and selective coding completed in line with the Strauss & Corbin approach.^15^ Open coding allowed for the identification of key concepts, axial coding involved linking of concepts into initial sub‐categories according to their commonalities, and selective coding facilitated the formalising of these associations into theoretical categories, identifying a core category. Discussion between the research team and the CEI groups confirmed interpretation.

### 2.1 Community engagement and involvement

Community engagement and involvement (CEI) groups in each country were integrated into the research from the design stage; they provided additional input commenting on study documents, providing support in introducing potential participants to researchers and reviewing study findings, adding confirmability.

## 3 RESULTS

Interviews were conducted with 124 participants: 33 women, 18 male partners, 19 family members, 29 health workers and 25 community leaders, across the three countries. Demographic data are presented in Table 1. The majority of women participants were multigravida, with 16 participants experiencing antepartum and 17 intrapartum stillbirth. This reflects the position in sub‐Saharan Africa where approximately half of stillbirths occur during labour.^3^ All women experienced stillbirth after 28 weeks’ gestation, as per the WHO definition. Participant views were similar across all settings, regardless of parity and gestation, and are presented together under the following key themes; ‘*needing to know versus fear of knowing’*, ‘*minimal harm’* and ‘*negotiating the decision,’* leading to a core category of ‘*moving towards understanding’*. This core category represents the needs and willingness of parents and health professionals to know the causes of stillbirth and parents’ preparedness to accept investigations. However, this was tempered by tradition and culture, which crosscut all themes, acting as a barrier to progress in understanding the causes of stillbirth.

**TABLE 1:** Participant demographics

	Zambia					Tanzania					Malawi				
	Women *n* = 9	Partner *n* = 3	Family *n* = 3	HCP *n* = 9	CL *n* = 4	Women *n* = 16	Partner *n* = 11	Family *n* = 8	HCP *n* = 10	CL N = 12	Women *n* = 8	Partner *n*N = 4	Family *n* = 8	HCP *n* = 10	CL *n* = 9
Age Median (range)	35 (27–38)	34 (28–39)	57 (56–64)	37 (30–50)	41 (29–62)	35 (21–40)	41 (27–54)	46 (35–60)	35 (27–59)	52 (38–63)	23 (18–29)	29 (20–41)	47 (39–57)	31 (26–39)	51 (40–70)
Marital status															
Married	9	3	1	5	4	14	11	7	9	10	6	3	5	5	8
Single	0	0	0	3	0	0	0	0	1	0	2	1	0	5	0
Widowed	0	0	1	1	0	0	0	1	0	0	0	0	3	0	1
Divorced/ separated	0	0	1	0	0	2	0	0	0	2	0	0	0	0	0
Education															
None	1	0	1	0	0	0	1	0	0	1	0	0	1	0	1
Primary	4	2	2	0	1	8	5	6	0	7	3	2	6	0	3
Secondary	3	1	0	0	2	6	5	2	0	2	5	2	1	0	1
College/university	1	0	0	9	1	2	0	0	10	2	0	0	0	10	4
Religion															
Christian	9	3	3	8	4	16	10	7	10	12	7	3	8	9	8
Muslim	0	0	0	0	0	0	1	0	0	0	1	1	0	1	1
None	0	0	0	1	0	0	0	1	0	0	0	0	0	0	0
Employment															
None/housewife	5	0	0	0	0	2	1	2	0	0	3	0	0	0	0
Employed/business	3	3	3	0	0	14	10	6	0	0	5	4	8	0	0
Nurse	0	0	0	0	0	0	0	0	7	0	0	0	0	0	0
Nurse/midwife	0	0	0	0	0	0	0	0	3	0	0	0	0	3	0
Midwife	0	0	0	7	0	0	0	0	0	0	0	0	0	4	0
Doctor	0	0	0	2	0	0	0	0	0	0	0	0	0	0	0
MO/CHW	0	0	0	0	0	0	0	0	0	0	0	0	0	3	0
Traditional leader	1	0	0	0	2	0	0	0	0	7	0	0	0	0	3
Religious leader	0	0	0	0	2	0	0	0	0	5	0	0	0	0	7
Previous births															
Primigravida	0					0					4				
Multigravida	9					16					4				

Abbreviations: CL, community leader; HCP, healthcare professional.

### 3.1 Needing to know versus fear of knowing

The majority of women and partners would welcome investigations to determine the cause of death of their baby, frequently citing understanding as potentially improving the outcomes of future pregnancies.

‘Absolutely. I would agree so that I know what caused my baby’s death. If at all in future, I plan to have another baby then I need to take precautions.’ (Woman 7, antenatal stillbirth, Tanzania)

Some women considered possible benefits for others, demonstrating altruism despite their own difficulties.

‘Because the problem that I may face, it may also happen to another woman. So, it may help them to see that the same thing has happened to another.’ (Woman 6, intrapartum stillbirth, Malawi)

This was echoed by community leaders who believed there were benefits to the community in reducing stillbirth and health workers who believed practice could be advanced with this knowledge. However, some women felt there would be no benefit, and potentially harm, in knowing the cause.

‘It won’t bring it back to life. The baby died between me and the nurses. Maybe I killed it. Knowing that it died out of my carelessness would devastate me.’ (Woman 12, intrapartum stillbirth, Tanzania)

The element of blame attribution resulting from investigations was a potential concern for many. Some family members believed that knowing the cause would place the blame on health workers, vindicating the family. Conversely, community leaders felt that knowing the cause might reduce conflict between women, families and health workers.

‘It means to remove the conflict [and] misunderstanding between the mother and the nurses to know the cause of the death, because when the investigations are done, means the source and the cause of death will be known.’(Religious leader 4, Tanzania)

The potential for the women to receive blame and stigma remained, with dissonance between the potential for investigations exonerating the women and others ‘finding her out’.

‘If the mother caused the stillbirth, this will be a learning lesson for her.’ (Traditional leader 4, Tanzania)

### 3.2 Minimal harm

There was an overall acceptance of the concept of investigations, but some techniques were more acceptable than others (see Table 2). Although the infant was viewed as a body, rather than a person, many parents and family members were reluctant to accept invasive measures, particularly those involving cutting, on the basis that it may ‘hurt’ the baby. Non‐invasive techniques, such as skin swabs, and placental investigations were acceptable to almost all participants, and a needle biopsy was also acceptable to most (see Table 3). Community leaders felt it appropriate for parents to be guided by health professionals’ knowledge in their choice of investigation type and were more supportive of invasive investigations, such as postmortems. Health professionals were supportive of a range of investigations, should they be available, but understood they would not be acceptable to all women. However, health workers’ knowledge of investigations varied, and some did not feel equipped to give explanations.

**TABLE 2:** Acceptability of investigations by type

Postmortem/autopsy	*‘The cutting of a dead baby is what is not allowed and not acceptable in our culture.’ (woman 2, Malawi)* *‘I would not feel good. I already knew what killed him. Unless it was not known I would look at that option.’ (woman 14, Tanzania)* *‘I feel that a postmortem would just trouble my baby. The baby is already dead, why opening the baby to try and find out the case of death. I do not think that a postmortem would help.’ (woman 8, Zambia)* *‘I would have no problem with that. Investigating is knowing. We can only know the cause of death if we investigate… on the other hand, I know that this procedure is against our culture. Even when I would be willing to do a postmortem, my husband and other family members may not accept it.’ (woman 1, Zambia)* *‘I would have received it and accepted it. Even with an elderly person, there is a postmortem. With a child, they would have performed it to find out the cause of death. I would have accepted it.’ (partner 2, Zambia)*
Biopsy/bloods	*‘There is not a problem as long as they involved us. Also, as long as it is done at the hospital.’ (Woman 11, Tanzania)* ‘*I would not want my baby to be pricked with needles.’ (Woman 6, Zambia)* *‘I would agree, since it is something that is not breathing…. But, pricking it and withdrawing bloods or fluids from it, it’s hurting the body.’ (Family member 8, Malawi)* *‘They even prick us to take blood samples in order to do tests….I feel that there is no problem.’ (Religious leader 4, Malawi)*
Other	*‘If they are sensitised to the importance of doing tests by getting a piece from the placenta, they can be willing to do it.’ (Health professional 4, Zambia)* *‘I would accept a skin swab.’ (Woman 4, Tanzania)* *‘People are sometimes people are not comfortable with blood collections and getting a small part of their body. Some people believe in superstitions, especially like us here in the villages. A skin swab would be ideal.’ (Traditional leader 2, Zambia)*
Professional guidance	*‘I would still let the doctor decide on which methods will bring proper answers. I will let the nurses decide the best tests to be done. I cannot make decisions for them.’ (Traditional leader 4, Tanzania)* *‘I would have no objection because doctors are educated and know better’. (Woman 7, Malawi)*

**TABLE 3:** Participants’ potential acceptance of investigations by type

	Zambia			Tanzania			Malawi			Total
	Woman	Partner	Family	Woman	Partner	Family	Woman	Partner	Family	
	*n* = 9	*n* = 3	*n* = 3	*n* = 16	*n* = 11	*n* = 8	*n* = 8	*n* = 4	*n* = 8	*n* = 70
Postmortem										
Yes	1	3	0	4	8	1	3	1	0	21
No	6	0	2	1	1	4	4	0	4	22
Unsure	2	0	1	11	2	3	1	3	4	27
Biopsy										
Yes	6	3	2	11	9	5	3	1	4	44
No	3	0	1	1	1	3	4	3	4	20
Unsure	0	0	0	4	1	0	1	0	0	6
Skin swabs										
Yes	9	2	0	11	11	6	7	4	7	57
No	0	1	2	0	0	2	0	0	1	6
Unsure	0	0	1	5	0	0	1	0	0	7
Placental examination										
Yes	5	3	2	11	9	4	4	0	6	44
No	3	0	1	0	0	3	1	3	2	13
Unsure	1	0	0	5	2	1	3	1	0	13

‘I think if we are trained more about these options then I would be free to explain them. I don’t know anything about them.’ (Health worker 3, Tanzania)

Cultural beliefs were strong and reinforced by family members, who appeared less likely to support investigations.

‘The main influence is our culture. Stillbirths are associated to a bad spirit or curse from the family, as a result, any medical investigation is regarded as trying to annoy the spirit and it can strike again.’ (Family member 1, Zambia)

As well as feeling protective of their baby in terms of potential harm, trust in the facility and staff was important in the acceptability of investigations. Invasive procedures were most likely to be refused, as women were suspicious about the destination of samples taken from their baby, based on their understanding of local practices.

‘I cannot allow any procedures that involve cutting the baby… Who knows where they take those pieces they get from babies? Such procedures are against our culture and beliefs. We have heard of many stories of health workers selling body parts of human beings to ritualists. I cannot allow my baby to be subjected to such procedures.’ (Woman 2, intrapartum stillbirth, Zambia)

### 3.3 Negotiating the decision

All participants felt that the health worker caring for the woman should be the one to make the approach for consent to investigations. In making the decision itself, the majority of participants saw the couple as the sole or main decision makers. Health workers felt they should hold open conversations about potential investigations, but concerns regarding cultural influences, women’s emotional position, potential blame and their own knowledge impacted upon their confidence in doing this.

‘The mother’s reaction upon hearing of her baby’s death will prevent me from telling her about the postmortem…They have emotions. They may even accuse us of killing the baby. I think if we are trained more about these options then I would be free to explain them. I don’t know anything about them.’ (Health worker 3, Tanzania)

In making the decision, women also had to negotiate cultural influences and beliefs, which were often related to future pregnancy success, making women fearful of denying them.

‘According to our tradition, when I give birth to a stillborn baby, the baby should be buried in less than 24 hours. If the hospital is to start investigations, they are supposed to put the baby in the mortuary, which is not allowed in our tradition. If that is done, it means that I will never have any other child.’ (Woman 8, antenatal stillbirth, Zambia)

Families often reinforced these beliefs, and in some cases were instrumental in making decisions in areas where wider family involvement was expected. This affected the autonomy of the couple to decide and some parents felt the inclusion of family in decision making could be an issue, as they may be less supportive of investigations.

‘Family members can be a hindrance to this investigation, as they will think it is time wastage and wasting of money because anyway, you cannot bring back the baby.’ (Partner 3, Tanzania)

Partners accepted they had little knowledge of investigations and would be guided by health professionals in making the decision.

I will be listening to the doctor… because I have no knowledge about it. The doctor is the one who directs you. (Partner 8, Tanzania)

In making the decision, women were also mindful of the potential cost of the investigations and the fact that they may need to rely on others for financial support. This was particularly evident in Tanzania.

‘My sister, being informed, she wanted a postmortem to be done on the baby’s heart and other organs, but I told her that we could not afford the charges.’ (Family member 9, Tanzania)

The impact of potential cost was echoed by health workers in Malawi.

‘After the baby is born, sometimes we offer them to do histology of the placenta, like you take a biopsy and do a tissue diagnosis, but it’s not a free service. So far I haven’t come across anyone who can afford them.’ (Health worker 8, Malawi)

## 4 DISCUSSION

### 4.1 Main findings

This study has highlighted that most woman and partners want to understand the cause of the death of their baby. The main driving force for this is the desire to protect against stillbirth in future pregnancies. Some women, community leaders and health workers also understood the potential wider societal benefit from this knowledge. Parents in this study are open to a range of investigations from autopsy to non‐invasive options. However, this is tempered by the need to comply with cultural practices and beliefs, which form a potential barrier to parents’ acceptance of the offer of tests to discover why their baby died. These beliefs were often strongly reinforced by family members, who may act as a barrier to acceptance of investigations. Conversely, community leaders representing both traditional and religious groups appeared to be generally supportive of investigations and of the parents alone making decisions around this.

Women in this study shared similar concerns to women in high income settings, including prevention of further harm to the infant^7^, ^18^ and concerns of inappropriate use of infant organs. Although culture varied across the three countries, all participants were aware of local cultural norms and the potential influence on their choices. One practical application of culture was the requirement to bury the infant within 24 hours of birth, and many would not accept investigations which would necessitate delay. Other cultural beliefs focused on potential future ‘bad luck’ or further stillbirth if the baby is ‘disturbed’. Fear for women of additional blame as a result of investigations is a unique finding in this study. Whereas women in high‐income settings have reported that investigations alleviate anxiety,^19^ some participants in this study believed findings might expose women to further stigma and blame, by highlighting impacts of their pregnancy behaviour. A further important practical concern was financial outlay, with many parents unable to afford investigations even if they were available. Investigations related to the infant following stillbirth were very rare in these countries and some participants, including health workers, were unaware of the potential advantages and limitations of autopsy or non‐invasive examination.

### 4.2 Strengths and limitations

This is one of the first studies to explore the potential acceptability of investigations in sub‐Saharan Africa. The inclusion of partners, family, health professionals and community leaders has ensured contextual and comprehensive understanding, identifying areas to be considered, prior to potential implementation of wider investigations. As a grounded theory study, the findings are not generalisable, but they are likely to be transferable to other similar settings. The presentation of the findings to country stakeholder groups, CEI groups and in dissemination events (including health workers, women, families) established confirmability.

It is possible that some participants found it difficult to explore their views of investigations which are not routinely offered in these settings. This was mitigated by ensuring the research assistants were trained in describing the different approaches and the use of a standardised narrative to ensure all participants received the same information about each investigation.

### 4.3 Interpretation

The dissonance between parents wanting to understand the cause of death and cultural constraints was evident. The possibility of investigations was a positive concept for many who wanted to be able to move forward and actively protect against future pregnancy loss, which is particularly important, as prior stillbirth is an important risk factor for future pregnancy loss. Importantly, women in this study appeared less tolerant of a fatalistic acceptance of stillbirth than has been reported previously,^20^ indicating women may be becoming more actively engaged in their health. This understanding suggests that offering women choices in investigations may encourage them to be invested in their future health and empower them to make decisions around care.^21^ Such health empowerment is vital in improving maternal and neonatal outcomes and reducing stillbirth.^22^ However, tradition and culture were likely to have a negative influence on women’s decisions about investigations, regulating behaviour and choice.^23^ These cultural norms prevent women from exercising choice around the care of their stillborn baby and removes their ability to pursue clinical explanations. Women bound by cultural constraints are therefore unlikely to gain closure for the death, particularly in light of the limited community support available for bereaved parents.^2^, ^24^

The suggestion that understanding of cause of death may alleviate or determine blame requires consideration. Blame is associated with higher rates of psychological distress^19^ and reducing this would be beneficial.^6^ However, it is unclear how the outcome of investigations would be interpreted and whether this would, in fact, reduce the blame burden for many. Women may still suffer stigma and blame where the reasons for the death are not fully understood.^6^ In this study, some believed the results of investigations might shift the blame to health workers, absolving women of wrongdoing. However, this may have an impact on health workers wellbeing and potentially how they care for women.^25^ Health workers frequently experience blame for stillbirth, with little support,^1^ and increasing this may impact on how investigations are offered, becoming counterproductive. Prior to implementing investigations, it is important to consider how a no‐blame culture for women and health professionals could be developed in these settings, to ensure transparent discussions for individuals around the cause of stillbirth and future planning of care.

The availability of investigations requires consideration at local and national level, to ensure women are offered appropriate and effective choices. Health workers acknowledge the usefulness of postmortems, but this is not acceptable to all, either emotionally or financially. A compromise may be effective non‐invasive and potentially cheaper alternatives such as histopathological examination of the placenta.^5^, ^13^ The financial cost of stillbirth is considerable and reducing stillbirth through knowledge and treatment of cause will help to reduce this cost.^26^, ^27^ Consideration of free investigations for women experiencing stillbirth may be a positive forward move in addressing and preventing stillbirth while providing psychological closure and destigmatising treatment of women. This may also empower women to make decisions without relying on family for financial support.

Discussion of autopsy or investigation following stillbirth is sensitive and can be difficult for health workers and women to address.^8^ In settings where investigations are not common practice, particular care needs to be taken to ensure practitioner’ understanding of this unfamiliar concept. Participants in this study implicitly believed that any investigation would provide a definitive answer to cause of death. Hence, it is vital that discussions around investigations enable adequate understanding of the benefits and limitations, to prevent potential further emotional trauma for women.^18^ Parents also require clear information of the investigation on the body of their baby, given their continuing need to ‘protect’ the infant.^7^ Health workers will need to be equipped with the necessary skills and understanding of any investigations to be able effectively to discuss these with women, as evidence suggests they often lack training and skills in this area.^8^ Furthermore, health workers will be subject to similar cultural norms, which they will need to negotiate in facilitating these conversations in an open and non‐judgemental way.

## 5 CONCLUSION

Whilst women want to understand the cause(s) of their baby’s death, emotional, cultural and economic barriers exist. Non‐ or minimally invasive investigations may be the most acceptable options in these settings. However, certain considerations require addressing when introducing new investigations into already stretched health systems. To gain the widest reach and potentially improve care for women in subsequent pregnancies, facilitating factors would include community support, education of health workers, parents and communities, and affordability. Community leaders would be well placed to provide support for parents pursuing investigations, along with sensitisation of the community as a whole. Health workers would require adequate training and understanding of the types of available investigations, including their limitations, to be able to provide parents with the information they require to provide truly informed consent. The economic cost of investigations would also need to be considered in determining provision, to enable equity of access. It is important that where investigations are offered, women’s choice is paramount in accepting or declining these, and women should not face further stigma as a result of their decisions.

## AUTHOR CONTRIBUTIONS

CB and TL designed the study, with input into protocol development from AH, SV and BV. All authors contributed to specific country analyses (CB, VAD, TL, KT, KL, DK, HS, FK, CP) with CB, VAD and TL synthesising the overall findings. All authors interpreted the data. CB drafted the first version of the article. All authors (VAD, TL, KT, KL, DK, HS, FK, CP, AH, SV, BV) commented on drafts of the article and have read and approved the final version for publication.

## FUNDING INFORMATION

This research was funded by the National Institute for Health Research (NIHR) (16/137/53), a major funder of global health research and training, using UK aid from the UK Government to support global health research. The views expressed in this publication are those of the author(s) and not necessarily those of the NIHR or the UK Department of Health and Social Care.

## CONFLICT OF INTERESTS

None declared. Completed disclosure of interest forms are available to view online as supporting information.

## ETHICS APPROVAL

Ethical approval was gained from University of Manchester ethics committee (ref: 2019–7451‐11 496, 24/07/19), College of Medicine Research and Ethics Committee, Malawi (ref: P.09/19/2793, 22/11/19), the Catholic University of Health and Allied Sciences (CUHAS)/Bugando Medical Centre (BMC) Joint Ethical and Review Committee, Tanzania (ref: CREC/399/2019, 12/09/19) and National Health Research Authority and Ethics and Science Converge Institutional Review Board (ERES Converge IRB), Zambia (ref: 2019‐Aug‐020, 07/10/19).

## Supporting information


Table S1

ICMJE

ICMJE

ICMJE

ICMJE

ICMJE

ICMJE

ICMJE

ICMJE

ICMJE

ICMJE

ICMJE

ICMJE

ICMJE

## Data Availability

Data available on request from the authors.
