# Supplementary material for: ‘Moving towards understanding’, acceptability of investigations following stillbirth in sub‐Saharan Africa: A grounded theory study
Source: BJOG. 2022 Oct 21;130(1):59–67. doi: 10.1111/1471-0528.17319 (PMC10092083; doi:10.1111/1471-0528.17319)
Supplement: Supplementary file 1 — Table S1 [file BJO-130-59-s006.docx]

**Post-mortem explanation (to be used alongside topic guide)**

‘A post-mortem (also called an autopsy) is the medical examination of a body after death. Babies are examined by a pathologist, a doctor who specialises in identifying conditions that affect babies, and who examines babies to find out why they died.

The pathologist examines the outside of the body very carefully for any signs of abnormality, and then measures, weighs and examines all the internal organs in detail to try to find out why the baby died. The placenta (afterbirth) is also examined if it is available. The pathologist takes small samples of tissue from the organs for examination under a microscope, before returning all the organs to the body.’ [Adapted from Sands, 2017]
